# LGBT OLDER ADULTS IN RURAL SOUTHERN APPALACHIA: PERCEPTIONS OF CURRENT AND FUTURE FORMAL SERVICE NEEDS

**DOI:** 10.1093/geroni/igz038.2720

**Published:** 2019-11-08

**Authors:** Kelly A Williams, Emily Dakin

**Affiliations:** Appalachian State University, Boone, North Carolina, United States

## Abstract

Older sexual and gender minority adults in rural communities face challenges in accessing formal health, mental health, and long-term care services (Butler, 2017; Koch & Knutson, 2016; Stein et al., 2010). Formal service providers in rural Southern Appalachia are more likely to have conservative values that are closely linked to their religious beliefs (Keefe, 2005). Some may be opposed to gender non-conformity and same-sex relationships or marriage, making it wise for LGBT older adults in rural contexts to carefully select formal service providers and settings (Willging et al., 2006). Barriers to accessing formal services for LGBT older adults residing in rural contexts include few LGBT-inclusive service providers and facilities, transportation, cost, and health insurance (Butler, 2017). When faced with the prospect of long-term care, older LGBT adults are more likely to conceal their sexual or gender identity due to fears of being mistreated (Brotman et al., 2003). This session will present results of a qualitative study examining experiences, concerns, and recommendations regarding formal services among 11 LGBT older adults residing in rural southern Appalachia. Several of the participants described experiencing discrimination from local service providers. A number of participants were fearful about the perceived lack of LGBT-inclusive services in the area and expressed that they would consider leaving the area if their own or their partner’s health declined. Many participants expressed the need for local provider education about the needs of LGBT older adults. The presenters will discuss the implications for research and for health, social, and long-term care services.

